# Analysis of Resistance to Florfenicol and the Related Mechanism of Dissemination in Different Animal-Derived Bacteria

**DOI:** 10.3389/fcimb.2020.00369

**Published:** 2020-07-31

**Authors:** Peizhen Li, Tingyuan Zhu, Danying Zhou, Wei Lu, Hongmao Liu, Zhewei Sun, Jun Ying, Junwan Lu, Xi Lin, Kewei Li, Jianchao Ying, Qiyu Bao, Teng Xu

**Affiliations:** ^1^Key Laboratory of Medical Genetics of Zhejiang Province, Key Laboratory of Laboratory Medicine, Ministry of Education, Wenzhou Medical University, Wenzhou, China; ^2^School of Laboratory Medicine and Life Sciences, Wenzhou Medical University, Wenzhou, China; ^3^Institute of Biomedical Informatics, Wenzhou Medical University, Wenzhou, China; ^4^Research Center for Translational Medicine, Shanghai East Hospital, Tongji University School of Medicine, Shanghai, China; ^5^Institute of Translational Medicine, Baotou Central Hospital, Baotou, China

**Keywords:** flofenicol, *floR*, *cfr*, PFGE, MLST, genomics

## Abstract

Bacterial resistance to antibiotics has become an important concern for public health. This study was aimed to investigate the characteristics and the distribution of the florfenicol-related resistance genes in bacteria isolated from four farms. A total of 106 florfenicol-resistant Gram-negative bacilli were examined for florfenicol-related resistance genes, and the positive isolates were further characterized. The antimicrobial sensitivity results showed that most of them (100, 94.33%) belonged to multidrug resistance *Enterobacteriaceae*. About 91.51% of the strains carried *floR* gene, while 4.72% carried *cfr* gene. According to the pulsed-field gel electrophoresis results, 34 *Escherichia coli* were subdivided into 22 profiles, the genetic similarity coefficient of which ranged from 80.3 to 98.0%. The multilocus sequence typing (MLST) results revealed 17 sequence types (STs), with ST10 being the most prevalent. The genome sequencing result showed that the *Proteus vulgaris* G32 genome consists of a 4.06-Mb chromosome, a 177,911-bp plasmid (pG32-177), and a 51,686-bp plasmid (pG32-51). A *floR* located in a drug-resistant region on the chromosome of *P. vulgaris* G32 was with IS*91* family transposase, and the other *floR* gene on the plasmid pG32-177 was with an IS*CR2* insertion sequence. The *cfr* gene was located on the pG32-51 flanked by IS*26* element and TnpA26. This study suggested that the mobile genetic elements played an important role in the replication of resistance genes and the horizontal resistance gene transfer.

## Introduction

Florfenicol is a new type of broad-spectrum antibiotics of chloramphenicol for veterinary use, which was successfully developed in the late 1980's. It can also be recalled that thiamphenicol has the chemical formula C_12_H_14_C_l2_FNO_4_S. CH_3_SO_4_ replaced the -NO_2_ group of chloramphenicol and gave florfenicol an obvious advantage in safety and efficacy compared with chloramphenicol. The antimicrobial spectrum of florfenicol is extremely wide. It has an inhibitory effect not only on the majority of Gram-negative and Gram-positive bacteria, as well as part of chlamydia and rickettsia, but also on chloramphenicol-resistant bacteria (Sams, 1995; Fang et al., 2020). Thus, florfenicol has been applied gradually across the world since it was launched in Japan in 1990. It is widely used in veterinary clinics, as a veterinary drug and feed additive, for its good therapeutic efficacy to bacterial diseases of pigs, cattle, poultry, and fish. However, with the irrational usage of florfenicol in clinical veterinary, serious resistance problems have emerged. Recently, many florfenicol-resistant bacteria and florfenicol-related resistance genes have been isolated from various animals. In 1996, florfenicol-resistant gene *pp-flo* was isolated from the multidrug-resistant plasmid R of the fish *Pasteurella* in Japan (Kim and Aoki, 1996). Then, *floR* was detected in the plasmids and the chromosomes of *Escherichia coli* from cattle, poultry, and pigs (Arcangioli et al., 1999; Bolton et al., 1999). In addition, it was also found in the IncC plasmid R55 of *Klebsiella pneumoniae* (Cloeckaert et al., 2001), a closely related plasmid of *Salmonella newport* (Meunier et al., 2003), and the chromosomes of *Vibrio cholerae* (Hochhut et al., 2001). Resistance to florfenicol in clinical settings has also been observed in human-originated *E. coli* isolates (Fernández-Alarcón et al., 2011). According to the study, the FloR protein contains 12 hydrophobic transmembrane regions belonging to the main susceptibility factor superfamily, which is located on the bacterial membrane and plays a significant role in the resistance to antibacterials (Hayes et al., 2006). In 2000, *cfr* gene was cloned from *Staphylococcus sciuri* by S. Schwarz, mediating the resistance of *S. sciuri* to florfenicol. The sequence analysis results suggested that *cfr* is a new type of florfenicol resistance gene (Schwarz et al., 2000). There was no homology between *cfr* and *floR* (floR-like), and it also does not share any homology with the amino acid sequence of chloramphenicol acetyltransferase. A novel gene *fexA* which encoded an efflux pump in Gram-positive coccus *Staphylococcus lentus* was located on a 34-kb plasmid PSPCFS2 that conferred resistance to florfenicol and chloramphenicol (Fessler et al., 2010). With in-depth studies, many florfenicol-associated resistance genes have been discovered constantly, such as the phenicol-specific exporter genes *fexB, pexA* (Liu et al., 2012), AcrAB-Tok multidrug efflux system *tolC* gene, *acrB* gene (Lee et al., 2000), and novel ATP-binding cassette (ABC) transporter gene *optrA* (Wang et al., 2015). Most of the genes co-existed with the bacterial mobile genetic elements, including plasmids, transposons, or integrons, which contributed to the rapid spread of florfenicol resistance genes to numerous bacterial species through horizontal gene transfer (HGT).

Therefore, the objective of this study was to investigate the prevalence and the distribution of florfenicol-related resistance genes in bacteria isolated from chicken farms and goose farms in Zhejiang Province. We also focused on the characterization of the potential novel mobile genetic elements associated with the dissemination of florfenicol-related resistance genes through HGT. It will be helpful to identify the major resistance determinants responsible for this resistance and provide evidence for the rational use of drugs in veterinary medicine.

## Materials and Methods

### Bacterial Strain Collection and Identification

A total of 32 samples were collected from the feces of chickens and geese from three different chicken farms and one goose farm in three regions of South China in 2015. The samples were processed as previously described (Miranda and Zemelman, 2002; Miranda and Rojas, 2007), and florfenicol-resistant strains were recovered by a spread plate method using Tryptic soy agar (Difco, Franklin Lakes, NJ, USA) containing 30 μg/ml of florfenicol (Schering-Plough, Kenilworth, NJ, USA), (Schwarz et al., 2004). The strains were identified by pathogen isolation, morphologic observation, biochemical reaction, and sequencing of 16S rDNA amplified by polymerase chain reaction (PCR). The purified strains were stored at −80°C in Tryptic soy broth (Difco) supplemented with 20% glycerol and florfenicol (30 μg/ml), (Fernández-Alarcón et al., 2010).

### Antibiotic Susceptibility Testing

The minimum inhibitory concentrations (MICs) of florfenicol, tetracycline, chloramphenicol, cefuroxime sodium, enrofloxacin, levofloxacin, gentamicin, ampicillin, imipenem, ceftazidime, ciprofloxacin, colistin, and nalidixic acid were determined using the standard agar dilution method. The susceptibility results were categorized according to the guidelines from the Clinical and Laboratory Standards Institute (CLSI) 2017 (CLSI document M100-S27, 2017). *E. coli* ATCC25922 and one *floR* gene-positive *K. pneumoniae* strain1341 from our collection were used as controls.

### Detection of the Florfenicol Resistance Genes

The genes were detected by PCR. Genomic template was extracted using a bacterial genomic DNA extraction kit (Takara), following the manufacturer's instructions. The final 30 μl of PCR mixture containing ExTaq Premix (Takara), DNA template, and a pair of primers of the florfenicol resistance genes (floR, fexA, fexB, cfr, and optrA) was prepared for PCR (Cloeckaert et al., 2000; Zhang et al., 2011; Gómez-Sanz et al., 2013). The mixture was initially denatured at 94°C for 5 min, followed by 30 cycles of 94°C for 40 s, 55°C for 45 s, and 72°C for 50 s and was then elongated at 72°C for 10 min (Table 1). The PCR products were detected by electrophoresis in 1.5% agarose gels.

**Table 1:** PCR primers used in this work.

**Gene**	**Sequence (5^′^ → 3^′^)**	**Length (bp)**	**(Tm =°C)**
*floR*	F: ACGTTTATGCCAACCGTCCT	398	55
	R: CATTACAAGCGCGACAGTGG		
*fexA*	F: TTTCGCTGTTCTTGTGTTCG	358	59
	R: ACCTTGGAAAATCCCCATTC		
*fexB*	F: ACTGGACAGGCAGGCTTAAT	320	59
	R: CCTGCCCCAAGATACATTGC		
*optrA*	F: AGGTGGTCAGCGAACTAAGATAG	338	64
	R: TCAATCAAGCGTGTAATCCTTTCA		
*cfr*	F: GGGAGGATTTAATAAATAATTTTGGAGAAACAG	580	62
	R: CTTATATGTTCATCGAGTATATTCATTACCTCATC		
*adk*	F: ATTCTGCTTGGCGCTCCGGG	583	54
	R: CCGTCAACTTTCGCGTATTT		
*fumC*	F: TCACAGGTCGCCAGCGCTTC	806	54
	R: GTACGCAGCGAAAAAGATTC		
*purA*	F: CGCGCTGATGAAAGAGATGA	816	54
	R: CATACGGTAAGCCACGCAGA		
*icd*	F: ATGGAAAGTAAAGTAGTTGTTCCGGCACA	878	54
	R: GGACGCAGCAGGATCTGTT		
*gyrB*	F: TCGGCGACACGGATGACGGC	911	60
	R: ATCAGGCCTTCACGCGCATC		
*mdh*	F: ATGAAAGTCGCAGTCCTCGGCGCTGCTGGCGG	932	60
	R: TTAACGAACTCCTGCCCCAGAGCGATATCTTTCTT		
*recA*	F: CGCATTCGCTTTACCCTGACC	780	58
	R: TCGTCGAAATCTACGGACCGGA		
*floR*	R: CGAATTCATGACCACCACACGCCC	1,396	56
	F: AGGATCC TTAGACGACTGGCGACTTCTC		
*cfr*	R: ACCCGGG ATGCAAATTGTGAAAGGATGAAAG	1,073	57
	F: AGCGGCCGCCTATTGGCTATTTTGATAATTAC		

*The underlines for floR represent EcoR I and BamH I. The underlines for cfr represent Ava I and Not I*.

### Pulsed-Field Gel Electrophoresis

The chromosomal DNA of E. coli strains carrying the floR gene was digested with the restriction enzyme XbaI and then subjected to pulsed-field gel electrophoresis (PFGE) analysis (Xia et al., 2010). Salmonella enterica serovar Braenderup H9812 genome was used as a size standard. The gels were then electrophoresed in a CHEF-Mapper system (Bio-Rad, USA) in 1% pulsed-field certified agarose (Bio-Rad, USA) with 0.5× tris-borate and ethylene diamine tetraacetic acid as the running buffer at 14°C and 6 V/cm for 20 h. The pulse time ramped up from 5 to 20 s. Images were captured with Gel Doc system (Bio-Rad, USA).

### Multilocus Sequence Typing

E. coli DNA was extracted using a bacterial genomic DNA extraction kit (Takara, Dalian, China) following the manufacturer's instructions. Multilocus sequence typing (MLST) was performed using seven conserved housekeeping genes (adk, purA, fumC, mdh, icd, gyrB, and recA; https://pubmlst.org). The mixture was initially denatured at 94°C for 5 min, followed by 30 cycles of 94°C for 40 s, 54°C for 45 s, and 72°C for 50 s and was then elongated at 72°C for 10 min. The annealing temperatures were set at 54°C for adk, fumC, purA, and icd, 58°C for recA, and 60°C for gyrB and mdh. The amplified fragments for all loci were sequenced. The allelic profiles and sequence type determinations were performed according to the E. coli MLST website scheme. The MLST data were analyzed by using the eBURST algorithm (http://eburst.mlst.net), which assesses the relationship within clonal complexes.

### DNA Sequencing and Analysis

Genomic DNA was isolated from bacterial cells grown overnight in Luria–Bertani broth at 37°C. The DNA was isolated by using QIAmp DNA mini kit (Qiagen, Valencia, CA, USA). The DNA concentrations and purity were determined by measuring the absorbance at 260 and 280 nm in a Nanodrop 2000 spectrophotometer. The genomic DNA of Proteus vulgaris G32 was sequenced by Pacific sequencing technology. The assembly of the sequence was performed with the help of SOAPdenovo v2.04, Celera Assembler 8.0 (Zhao et al., 2010), and Gap Closer v1.12 (Mendes et al., 2008). We used the Glimmer software to predict protein-coding genes with potential open reading frames (ORFs), (Delcher et al., 2007). RNAmmer and tRNAscan-SE were utilized to identify rRNA and tRNA genes, respectively (Lowe and Eddy, 1997; Lagesen et al., 2007). Gview was used to construct the basic genomic features (Petkau et al., 2010). BLASTX was used to annotate predicted protein-coding genes against the public protein database with an e-value threshold of 1e-5. Genome-wide identification of restriction–modification systems was conducted by using BLASTP searching against Rebase with >50% amino acid identity and >50% query coverage after all ORFs were theoretically translated (Roberts et al., 2015). The complete nucleotide sequences of the chromosome and the two plasmids of P. vulgaris G32 have been submitted to GenBank, and the accession numbers of the chromosome, pG32-177 and pG32-51, are CP053371, CP053372, and CP053373, respectively.

### Functional Analysis

The DNA fragments containing the floR and the cfr genes were amplified by PCR using the primers (Table 1). The complete ORF fragment of the PCR product was cloned into a pMD19 vector (TaKaRa, Dalian, China). The recombinant clones were picked and sequenced. The recombinant plasmid was digested with restriction endonucleases, and the ORF fragment was recovered and further cloned into a pET28a vector (TaKaRa, Dalian, China). Finally, the recombinant plasmids were transformed into the host strain BL21. The minimal inhibitory concentrations of antibiotics were determined by the agar dilution method for the recipient strains BL21, BL21[pET28a-floR], and BL21[pET28a-cfr] in accordance with the guidelines of the Clinical and Laboratory Standards Institute (CLSI, 2017).

## Results

### Antibiotic Resistance of the Strains

A total of 106 florfenicol-resistant Gram-negative bacilli were analyzed in this study. Most of the isolates were demonstrated to be *E. coli* (91 strains). The remaining isolates belonged to genera or species of *Bacillus proteus* (six strains), *Shigella* (five strains), *K. pneumoniae* (two strains), and *Salmonella* (two strains). The antibiotic susceptibility tests showed that most of them (100, 94.33%) were resistant to three commonly used veterinary antibiotics, and more than a half of the strains were resistant to more than seven antibiotics (55, 51.89%, Table 2). The percentages of strains resistant to florfenicol, ampicillin, enrofloxacin, tetracycline, chloramphenicol, cefuroxime sodium, gentamicin, levofloxacin, nalidixic acid, ceftazidime, and ciprofloxacin were 100% (106/106), 98.11% (104/106), 93.39% (99/106), 91.51% (97/106), 88.68% (94/106), 84.91% (90/106), 61.32% (65/106), 52.83% (56/106), 48.11% (51/106), 41.15% (44/106), and 36.68% (39/106), respectively. The lowest levels of resistance were found for colistin (3.77%, 4/106) and imipenem (0.94%, 1/106), (Table 3).

**Table 2:** Number, multi-resistance rate of resistant strains, and distribution of antimicrobial resistance genes.

**Strains**	**Farm A (H1-22)**	**Farm B (H23-46)**	**Farm C (H47-71)**	**Farm D (G1-38)**	**Total**	**Multi-resistance rate (≥3)**	**Multi-resistance rate (≥7)**	**floR**	**cfr**
E. coli	19	19	21	32	91	95.60%	57.14%	95.60%	2.20%
B. proteus	0	2	1	3	6	66.67%	33.33%	66.67%	16.67%
Shigella	1	2	2	0	5	100%	0	80.00%	20.00%
Salmonella	1	0	0	1	2	100%	0	50.00%	50.00%
K. pneumoniae	0	0	1	1	2	100%	50%	100.00%	0
Total	21	23	25	37	106	94.33%	51.89%	91.51%	4.72%

**Table 3:** Antimicrobial resistance profiles of resistant strains.

**Antibiotic**	**CLSI breakpoint interpretation**			**MIC (mg/ml)**					
	**S**	**I**	**R**	**MIC_**50**_**	**MIC_**90**_**	**P. vulgaris G32**	**BL21[pET28a-floR]**	**BL21[pET28a-cfr]**	**BL21**
CXM	2.83%	12.26%	84.91%	1,024	2,048	2,048	16	16	16
AMP	0.94%	0.94%	98.11%	512	>1,024	1,024	2	2	2
CAZ	54.72%	4.72%	41.15%	16	32	2	0.5	0.5	ND
IMP	98.11%	0.94%	0.94%	<1	<1	4	ND	ND	ND
FFC	0	0	100.00%	128	256	256	256	64	16
CHL	3.77%	7.55%	88.68%	64	128	64	64	64	4
ENR	1.87%	4.72%	93.39%	>2,048	>2,048	>2,048	0.5	0.5	0.5
LVFX	40.57%	6.60%	52.83%	0.5	4	2	<2	<2	<1
CIP	61.32%	1.89%	36.68%	2	16	16	<0.5	<0.5	<0.5
NAL	47.17%	4.72%	48.11%	8	512	1,024	4	4	2
TET	3.77%	4.72%	91.51%	128	256	16	4	4	4
GEN	36.79%	1.89%	61.32%	16	128	16	2	4	2
CL	95.53%	0.94%	3.77%	<0.5	2	1	<0.5	<0.5	<0.5

*FFC, florfenicol; CHL, chloramphenicol; TET, tetracycline; LVFX, levofloxacin; GEN, gentamicin; AMP, ampicillin; IMP, imipenem; CAZ, ceftazidime; CIP, ciprofloxacin; CXM, cefuroxime sodium; ENR, enrofloxacin; CL, colistin; NAL, nalidixic acid*.

### Detection of Florfenicol Resistance Genes

We screened the florfenicol resistance genes from above 109 strains *via* PCR. About 91.51% (97/106) of the strains showed *floR* gene-positive results, 4.72% (5/106) showed *cfr* gene-positive results, while no other known florfenicol-resistant genes (*fexA, fexB*, and *optrA*) were detected (Table 2). Among them, only one isolate was positive for both *floR* and *cfr*, which was *P. vulgaris* G32 from the goose farm.

### Pulsed-Field Gel Electrophoresis Results

**Figure 1:**
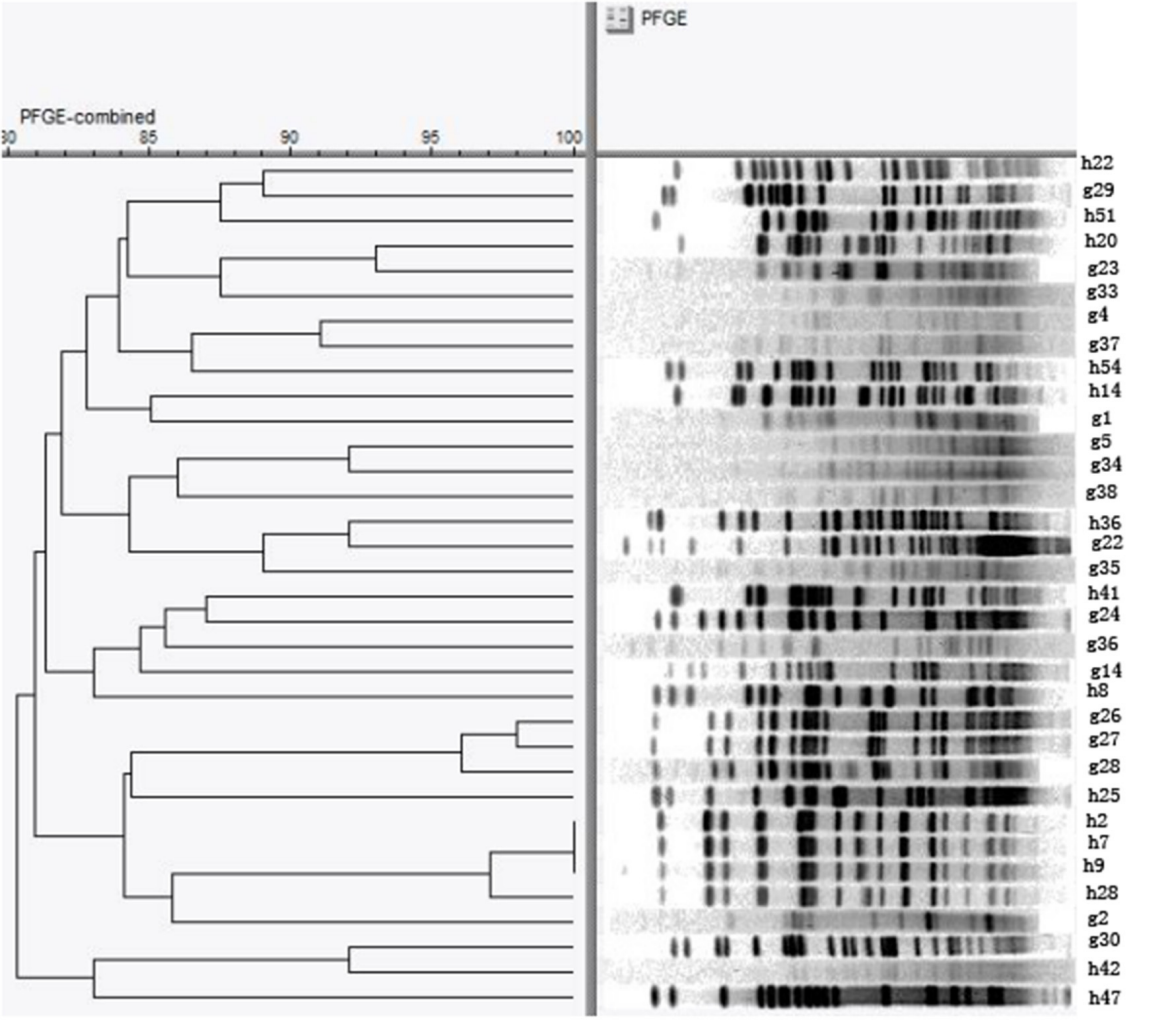
Pulsed-field gel electrophoresis (PFGE) pattern of 34 *E. coli* resistant to more than seven drugs. The chromosomal DNA of 34 *E. coli* isolates carrying the *floR* genes were digested with the restriction enzyme *Xba*I and then subjected to PFGE analysis.

We conducted a further epidemiological study on *E. coli*, the most common florfenicol-resistant bacteria in this region. A total of 34 *floR*-positive *E. coli* strains which are resistant to more than seven drugs from four farms were typed by PFGE. Bands with sizes ranging from 20 to 700 kb were obtained. The number of electrophoretic bands was between 15 and 28. A restriction map containing 22 bands was obtained when a cutoff of identity was set at 100% (Figure 1). By UPGMA cluster analysis, the genetic similarity coefficient ranged from 80.3 to 98.0%; each band type included one to two strains. The similarity coefficient of *E. coli* H20 from chicken farm B and *E. coli* G23 from the geese farm was 93%. In addition, the similarity coefficient among *E. coli* H2, H7, H9 (farm A), and H18 (farm B) was 98%. These results indicated that the dissemination of *floR* in this district was most possibly mediated by HGT.

### Multilocus Sequence Typing Results

**Figure 2:**
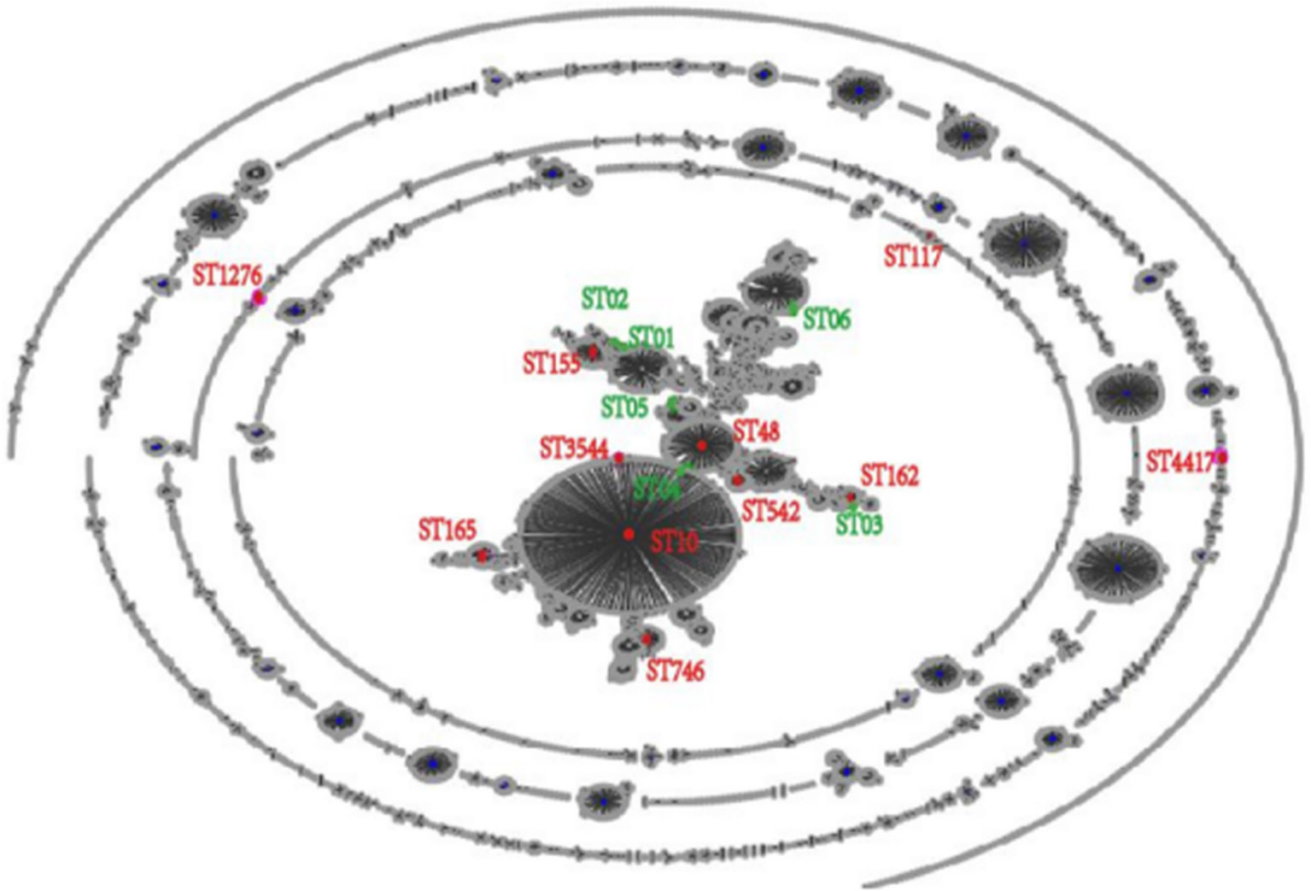
EBURST result of 6,475 sequence types (STs) in Pubmlst/*E. coli* database. All the 6,475 STs were clustered into 17 STs.

Based on the abovementioned PFGE results, a total of 18 strains from the dominant clusters were selected for MLST analysis. As a result, 17 sequence types were identified, including six new STs (ST01n−06n), (Table 4 and Figure 2). ST10 and its single locus variants (SLVs) belonged to clonal complex 10 (CC10), which was the most commonly observed one. ST3544, ST10, and ST48 belonged to CC10, while ST155, ST01n, and ST02n were classified into CC155 (Figure 2). ST01n and ST02n were double-loci variants of ST155 because they had two different alleles. ST162 and ST03n belonged to CC469. ST03n was a SLV of ST469. ST165, ST05n, and ST06n belonged to CC65, CC206, and CC23, respectively. Furthermore, there were six singleton STs, including ST4417, ST1276, ST542, ST746, ST117, and ST04n, which could not be categorized according to any of the clonal complexes based on the MLST.

**Table 4:** Sequence types and allele numbers of *E. coli* isolates.

**Strain**	***Adk***	***fumC***	***purA***	***icd***	***gyrB***	***mdh***	***recA***	**ST**	**Clonal complex**
h2	6	6	401	18	7	18	6	ST 4417	
h7	457	65	5	1	9	13	6	ST 03n	CC469
h11	112	11	5	12	8	8	86	ST 542	
h14	6	4	14	16	24	8	14	ST 155	CC155
h16	9	11	4	8	8	8	2	ST 3544	CC10
h22	603	4	4	16	24	8	104	ST 01n	CC155
h27	10	11	4	8	8	8	2	ST 10	CC10
h38	9	65	5	1	9	13	6	ST 162	CC469
h41	17	231	167	198	7	157	2	ST 1276	
h48	17	231	167	198	7	157	2	ST 1276	
h54	457	4	4	16	24	8	104	ST 02n	CC155
g1	10	27	5	10	12	8	2	ST 165	CC65
g4	603	11	4	-	8	8	2	ST 04n	
g5	603	7	5	1	8	18	2	ST 05n	CC206
g14	6	11	4	8	8	8	2	ST 48	CC10
g28	10	7	4	8	12	8	2	ST 746	
g33	20	45	41	43	5	32	2	ST 117	
g38	306	4	12	1	20	12	7	ST 06n	CC23

### General Features of *P. vulgaris* G32 Genome

**Figure 3:**
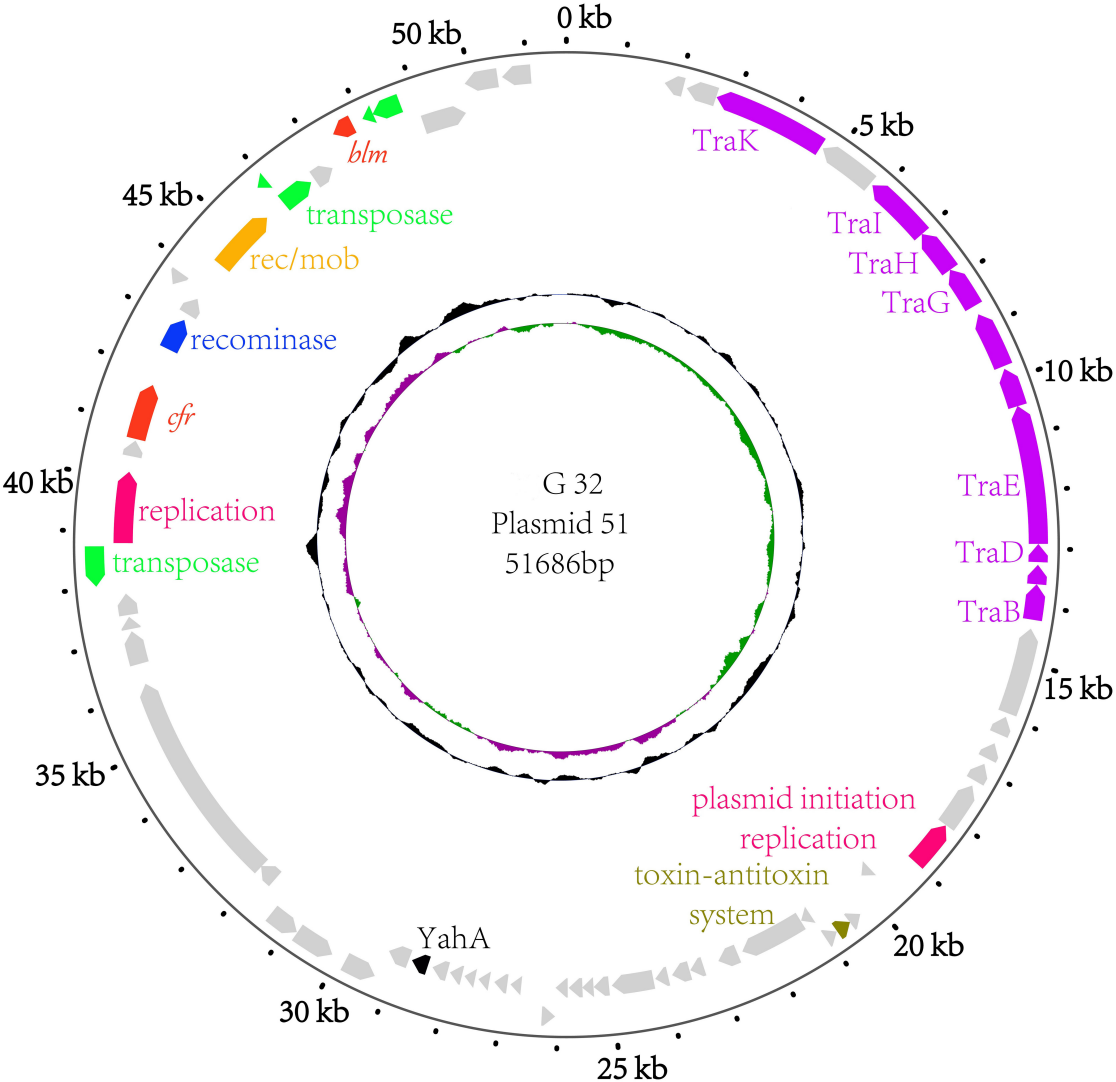
The circular map of the pG32-51 genome. Counting from outside toward the center, the first circle refers to the position in base pairs. The second circle consists of two-direction arrows which indicate the position of the gene and the marked genes encoded on the leading strand (outwards) or lagging strand (inwards). The different function genes are shown in different colors: red, drug-resistance related genes; purple, conjugation and transfer; green and yellow, transposase/insertion sequences; rose red, replication; blue, recominase; silver, unknown function genes. The third circle shows the GC skew (G–C/G+C), with a positive GC skew toward the outside and a negative GC skew toward the inside. The fourth circle shows the GC content with an average of 50%, whereby a G+C content of more than 50% is shown toward the outside and a G+C content of <50% toward the inside.

**Figure 4:**
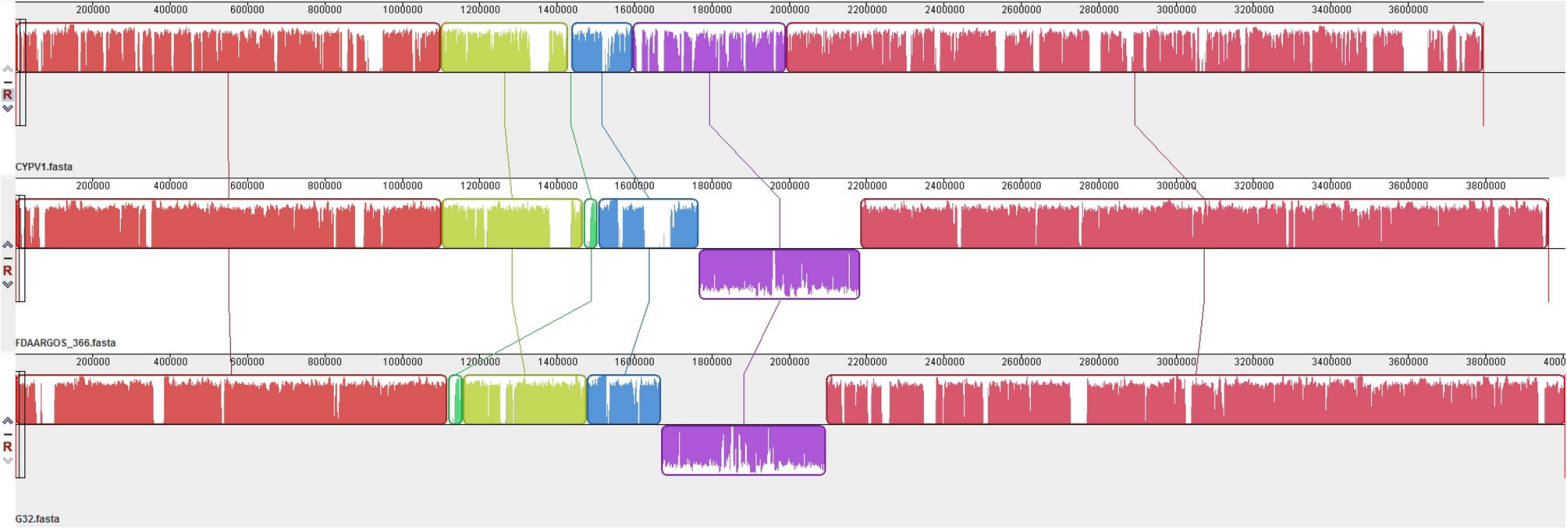
Genome structure comparison of *P. vulgaris* G32 with CYPV1 and FDAARGOS_366. The corresponding blocks from these three strains are shown according to the gene content or sequence similarities. The blocks below the line in strains CYPV1 and FDAARGOS_366 genome indicated that the sequence in the block was in a reverse direction compared to the corresponding region in *P. vulgaris* G32. The denser and higher lines represented more genes and higher similarities between the sequences.

The results of PCR screening showed that the *P. vulgaris* G32 also carried *floR* and *cfr* resistance genes. It is rarely reported in *B. proteus*. The genome sequencing result showed that the *P. vulgaris* G32 genome consists of a 4.06-Mb chromosome with an average GC content of 38.1% encoding 3,590 open reading frames (Table 5), a 177,911-bp plasmid (pG32-177) encoding 251 ORFs, and a 51,686-bp plasmid (pG32-51) encoding 80 ORFs (Figure 3). A total of 34 antibiotic resistance genes were identified, of which eight were encoded on the chromosome, 24 (including *cfr, floR, sul2*, aminoglycoside resistance genes, β-lactamase genes, and so on) on pG32-177, and three on pG32-51. The co-linear analysis of the genomes among sequenced *P. vulgaris* G32, *Proteus mirabilis* CYPV1, and *P. vulgaris* FDAARGOS-366 in GenBank showed a high collinearity among the three chromosomes, except that partial inversion, insertion, and deletion were observed (Figure 4).

**Table 5:** Basic characteristics of *P. vulgaris* G32 genome.

**Statistics**	**Chromosome**	**pG32-177**	**pG32-51**
Gene number	3,590	251	80
Size of genome (bp)	4,006,607	177,911	51,686
G+C content (%)	38.10	35.12	43.99
Coding region size (bp)	3,391,731		
Coding region/genome length (%)	84.65		
Average gene length (bp)	944		
Intergenic region size (bp)	614,876		
Ratio of intergenic region (%)	15.35		
Number of rRNA operons	22		
Number of tRNA genes	83		
Plasmid	2		

### Analysis of the Regions of the Chromosome Containing *floR* Genes

**Figure 5:**
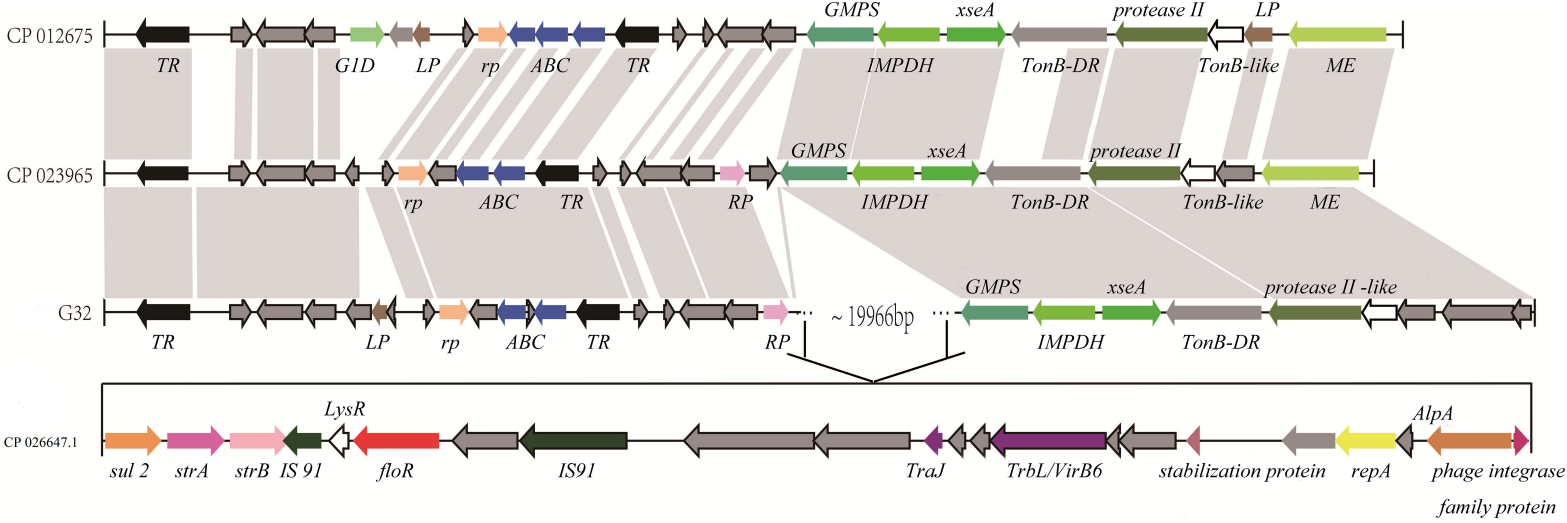
Comparison of the floR gene regions on the chromosome of P. *vulgaris* G32, *Proteus* CYPV1 (accession number CP012675), *Proteus* FDAARGOS-366 (accession number CP023965), and *V. cholerae HC1037* (accession number CP026647.1). The homolog genes are marked with the same color and lined together, respectively.

A florfenicol-related resistance gene *floR* is located in a drug-resistant region on the chromosome of *P. vulgaris* G32, which is highly homologous to the *Proteus* chromosomes from *Proteus* CYPV1 and *Proteus* FDAARGOS-366 in GenBank. The upstream of regions all contained transcriptional regulators, ABC super family, regulatory proteins, and so on, and the downstream region of a 13,425-bp segment of *P. vulgaris* G32 is homologous with the corresponding segment of *Proteus* FDAARGOS-366 and is different from *Proteus* CYPV1 slightly. The 19,966-bp region containing *floR* in the middle is a specific fragment of *P. vulgaris* G32. The sequence displayed that there is a pair of IS*91* family transposase locating upstream and downstream of the *floR* gene. Besides that, there are also TrbL/VirB6 associated with conjugation, transduction, and phage integrase family proteins existing in its downstream region. This gives reasonable explanations for the existence of this resistance segment. In other words, this segment was inserted into the chromosome with the help of integrase and transposase, and the insertion site was located between the repressor and the GMP synthetase. Transcriptional regulatory factor AlpA, regulatory protein RepA, and the stabilizing protein, which locates between TrbL/VirB6 and phage integrase family protein, also provide help for stable replication, transcription, and heredity to some extent. In addition, aminoglycoside-resistant genes *strA* and *strB* are also found in this region. Compared with the sequence of *V. cholerae* HC1037 (positions 95,522–102,832) from the clinical patients in the database, we can find that only part of the LysR open reading frame between IS*91* family transposase and *floR* is missing in the chromosome, and a *strA* gene between *sul2* and *strB* is different (Figure 5).

### Analysis of the Regions of the Plasmids Containing *floR* and *cfr* Genes

**Figure 6:**
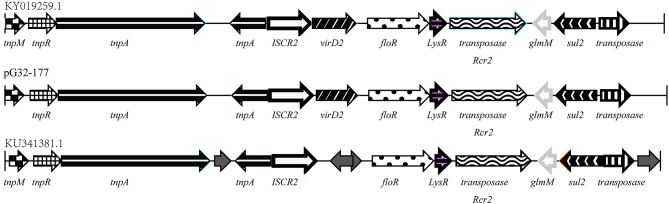
Comparison of the *floR* gene regions on the pG32-177, the sequenced plasmids of *E. coli* YJMC8 (accession number KY019259.1) and *E. coli* SHP45 (accession number KU341381.1).

In addition to the chromosome-encoded *floR, P. vulgaris* G32 also harbored another copy of *floR* gene on the plasmid pG32-177 with an IS*CR2* insertion sequence. A series of transposition-related genes including *tnpM, tnpR*, and *tnpA* was also observed near IS*CR2*, equipping this region with the autonomous transposition. This 13,177-bp segment is highly homologous with those of chicken-derived *E. coli* YJMC8 from Guangzhou, China, in 2017 and of porcine enteropathogenic *E. coli* SHP45 from Guangzhou, China, in 2016 (Figure 6).

**Figure 7:**
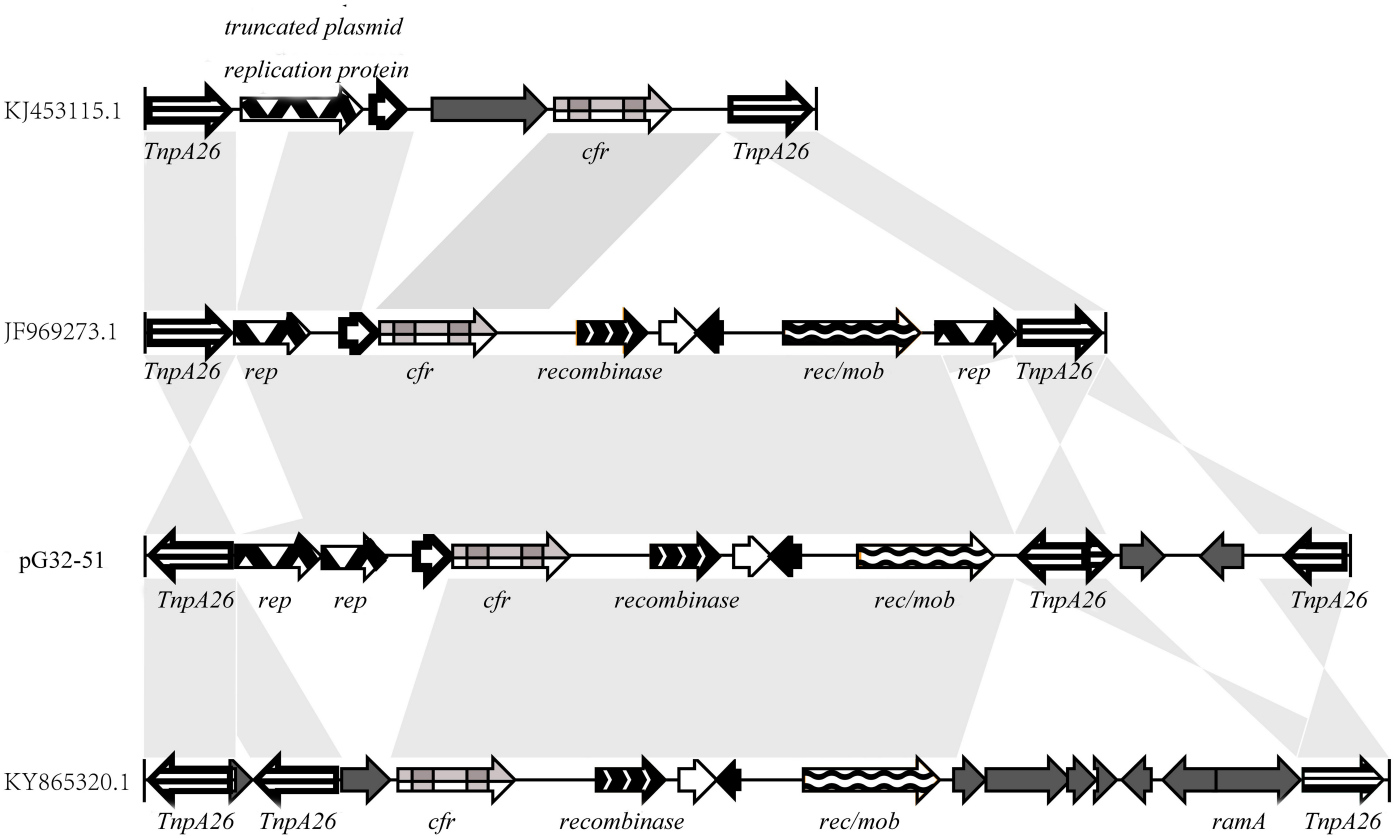
Comparison of the *cfr* gene regions on pG32-51, *E. coli* 8ZG12D (accession number KY865320.1), *E. coli* SH21G (accession number KJ453115.1), and the sequences of *P. vulgaris* strain PV-01 (accession number JF969273.1).

The *cfr* gene is located on pG32-51, flanked by two or three identical copies of the 820-bp IS*26* element encoding TnpA26 in the same orientation. Similar resistance gene regions have been detected in other sequenced plasmids of *E. coli* 8ZG12D, SH21G, and the sequences of *P. vulgaris* PV-01 (Figure 7). Compared with IS*26* insertion sequence, pG32-51 lacks a 1,030-bp segment whose function has to be further studied but has a segment of 4,150 bp which cannot be found in *E. coli* 8ZG12D. Rep, which exists at 3,927 bp downstream of *cfr* between rec/mob and tnpA26 in *P. vulgaris* PV-01, was located in the upstream of *cfr* immediately of tnpA26 of pG32-51. In addition, the resistant insertion segment of pG32-51 containing *cfr* follows a segment of 1,568 bp containing tnpA26. So, the main structure of the drug-resistant region is IS*26*-*cfr*-recombinase-rec/mob-tnpA26.

### Resistance Gene Cloning and Functional Analysis

We cloned the complete ORFs of both *floR* and *cfr* into pET-28a vectors and transformed them separately into *E. coli* BL21. The MICs of the antibiotics and the recipient controls against a group of antimicrobial drugs were detected (Table 3). BL21[pET28a-*floR*] and BL21[pET28a-*cfr*] showed resistance to florfenicol and chloramphenicol.

## Discussion

Man has developed new anti-bacterials to inhibit and eliminate the increasingly widespread and complex drug resistance of bacteria. However, new resistant strains are also appearing along with the clinical application of new antibiotics. The problem of florfenicol resistance is becoming serious; more and more drug-resistant bacteria have been found in the breeding environment and in animals (Bossé et al., 2015a). In 2000, White et al. studied the resistance of *E. coli* isolated from diarrhea cattle to chloramphenicol and florfenicol and got the result that 42 out of 44 strains (florfenicol MICs ≥ 16 μg/ml) carried *floR* gene (White et al., 2000). Kuo found that the resistance of *E. coli* isolated from pigs in Taiwan to florfenicol increased from 39.2% in 2003 to 78.3% in 2007, and the prevalence rate of *floR* gene showed a rising trend (Kuo et al., 2014). From a breeding farm in Heilongjiang Province, China, 60 strains of *E. coli* from swine were detected with positive rate as 50% of *cmlA* and 80% of *floR* (Zhao et al., 2017). In this study, we have examined the prevalence of florfenicol-related resistance genes in bacteria of animal origin. The results suggest that *floR* was the main epidemic resistance gene of florfenicol (91.74%); only five strains were positive to *cfr* (4.59%), and the rest of the resistance genes were not detected. In addition, the positive rate of *floR* gene was similar to the proportion of strains (MICs ≥ 32 μg/ml), agreeing with the results of Claudia et al. (2011). A PFGE analysis suggested that the degree of similarity of the bacterial diversity was low, and clone transmissions might exist in the multidrug-resistant *E. coli* strains carrying *floR* genes. In addition, the MLST analysis indicated that *E. coli* sequence types ST10, ST3544, and ST48 all belonged to clone complex 10 (CC10), the largest and most popular clone complex in the world (Shabana et al., 2013; Maluta et al., 2014). The detection of the common sequence type (ST10) in *E. coli* isolated from patients, farm workers, pigs, wild birds, and river water was reported, suggesting a possible transmission among animals, humans, and the surrounding environments (Fischer et al., 2017; Gomi et al., 2017). In other words, the popular strains in our research shared a high degree of homology with other major epidemic clones in the world.

There are many reasons for the emergence of resistance. Among them, the most important and widespread resistance mechanism is the acquisition and the transmission of various resistance genes. Most of the florfenicol resistance genes are located in the mobile plasmids and the transposons. The florfenicol resistance gene *pp-flo* (Kim and Aoki, 1996) was located in the multidrug-resistant R plasmid. An analysis of the plasmid pMBSFl with *floR* gene of the *E. coli* isolates from pigs (Blickwede and Schwarz, 2004) indicated that the flanking regions of *floR* gene are composed of three sequences from different sources. They are highly homologous to transposon Tn5393, plasmid with *floR* from *E. coli* 10660, and transposon Tn1721, respectively. The *floR* in pM3446F of these *Pasteurellaceae* plasmids was described as a transposable element encoding *floR*, transcriptional regulator *lysR*, and transposase *tnpA* genes initially (Bossé et al., 2015b). Zhang et al. confirmed that an IncA/C plasmid carrying the multiresistance gene *cfr* in a porcine *E. coli* strain was flanked by two copies of IS*256* (Zhang et al., 2014). Our study indicated that florfenicol resistance genes not only existed in the plasmids but also in the chromosomes of a bacterium. The *floR* gene in the chromosome of *P. vulgaris* G32 was mediated by the transposon IS*91*, which is different from the usual insertion sequence (Mataseje et al., 2014). Meunier et al. found the *floR* gene from bovine *E. coli*, which was shown to be associated with the insertion sequence IS*CR2* (Meunier et al., 2010). Similarly, our *floR* in pG32-177 is also next to an IS*CR2* insertion sequence, mediated by Tn*21*, indicating that the gene can be transferred with the composite transposons. The *cfr* gene in pG32-51 formed the structure of the composite transposon, recombinase-rec/mob-tnpA26, which can mediate the resistance genes mobile by homologous recombination or transferred by conjugation. That is consistent with the report of Doublet et al. (2005). Obviously, the molecular genetic background of florfenicol resistance genes is very complex. The transference of resistance plasmids in bacteria strengthens the resistance of drug-resistant bacteria and accelerates the spreading of resistance, and transposition links the resistance genes among chromosomes, plasmids, and phages and enriches the source of resistance plasmids.

The resistance problem of florfenicol has become a major problem in the development of animal husbandry, which needs to be tackled urgently. Resistance encoding genes determine the genetic complexity of the strains. The study on the prevalence and the environment of florfenicol resistance genes will contribute to the understanding of its origin, expression, and metastasis in molecular biology. It can also provide references for the rational usage of antibiotics and the further prevention of drug resistance in veterinary medicine.

## Data Availability Statement

The datasets presented in this study can be found in online repositories. The names of the repository/repositories and accession number(s) can be found in the article/supplementary material.

## Author Contributions

PL, QB, and TX designed the experiment. PL, TZ, DZ, WL, HL, and JuY performed experiments. XL, JiY, and TX contributed to analysis the experimental data. PL, TZ, and QB wrote the manuscript. PL, ZS, and JL supported and designed the project. KL, QB, and TX critically revised the manuscript. All authors read and approved the manuscript.

## Conflict of Interest

The authors declare that the research was conducted in the absence of any commercial or financial relationships that could be construed as a potential conflict of interest.
